# Editorial: Women in science - translational medicine 2021

**DOI:** 10.3389/fmed.2022.1021894

**Published:** 2022-09-07

**Authors:** Victoria I. Bunik, Claudine Habak

**Affiliations:** ^1^Faculty of Bioengineering and Bioinformatics, Lomonosov Moscow State University, Moscow, Russia; ^2^Department of Biokinetics, A. N. Belozersky Institute of Physico-Chemical Biology, Lomonosov Moscow State University, Moscow, Russia; ^3^Department of Biochemistry, Sechenov University, Moscow, Russia; ^4^Cognitive Neuroimaging Unit, Emirates College for Advanced Education, Abu Dhabi, United Arab Emirates

**Keywords:** diagnostic markers, diabetes, exaggerated inflammation, gender diversity in research, NAD in demyelinating diseases, chronic fatigue, low back pain

Gender diversity in research, ranging from team composition to methods and questions, can benefit scientific innovation (1) and impact (2). While the representation of women in early-career research roles is relatively equal to that of men, there is an under-representation in later-stage career roles (3), in corresponding authorship (4), and in lead authorship positions [first and last (5, 6)]. Globally, the pandemic has specific pressure on women's work, likely due to differences in family responsibilities (9–11). In science, women's research output has been affected disproportionately (7, 8), with a larger decrease in the number of pre-prints compared to men (12), and in the number of new projects and authorship—the medical literature included (7). Other special issues have addressed factors underlying gender gaps in science [e.g., (13)]. Here, we take action, by promoting the equal participation of women and men in science through the Research Topic “*Women in Translational Medicine*.” We create a space for translational studies by teams with female leaders, where the first and last authors are researchers identifying as female.

What comes across strongly in this collection, is the drive of female researchers to move translational medicine by reporting experimental data in unexplored and/or intricate fields (12 of the 13 papers consist of original research) and by highlighting novel approaches, such as a new paradigm in cancer diagnostics, presented by Glyn and Purcell in our only review of the series. The review exemplifies critical scientific discourse, providing a refreshing voice with strong and informative messaging: it demonstrates how whole-genome sequencing of microbiomes can discriminate between healthy and cancer subjects, even at early stages I and II. Advantages and limitations of this approach are compared to the diagnostic value of other types of DNA markers in cancer.

In the collection, over half the papers (7/13; including the review) are dedicated to diagnostic procedures for timely and effective intervention to treat complex pathologies and predict their course. One of the papers has acquired the highest number of views (>6,000 in 2 months post-publication) of all papers published in *Translational Medicine* over the past year. The work by González-Cebrián et al. offers a novel approach to diagnosing Chronic Fatigue Syndrome, which is a debilitating condition that can occur post-virally. The authors compared 15 female patients and 15 controls, and yet, reached significant discriminative power (healthy vs. patient), by analyzing 817 variables. As the microRNA profiles in the blood of patients are limited in diagnostic power, Raman micro-spectroscopy of blood extracellular vesicles along with other differences abundant in human patients are added to consideration. The authors stress that omics-based diagnostics should be enriched with statistical methods to reveal complex interactions among variables, which go beyond changes in individual markers. This approach is certainly key to personalized medicine.

The practical and financial costs of routinely measuring a wide array of data for single patients in clinical settings can be challenging, and several papers address the use of more feasible biomarkers. For example, biological markers of age correlate with the severity of chronic pulmonary obstructive disease (Campisi et al.), or can serve in organ transplantation (Pavanello et al.). These papers illustrate how determining biological age through cellular DNA methylation and telomere length can address various medical challenges. The use of another biomarker, a vitamin B3 derivative, NAD^+^, is presented by Balashova et al. Unlike previous findings in other human and animal tissues, NAD^+^ concentration in human blood does not decrease significantly with age. However, its decrease marks demyelinating neurological diseases and cardiopathologies, pointing to potential diagnostic applications of this test easily incorporated into routine blood analyses. Pia-Mara Wippert et al. show that readily available biomarkers, such as indicators of glucose metabolism in blood or the cortisol content in hair, complement psychometric tests in evaluating stress levels and long-term development of chronic low back pain. As the differential expression of symptoms may depend on individual levels of specific vitamins or other metabolic/hormonal indicators, devising and introducing biochemical tests in clinical practice improves the interpretation of symptoms and disease treatment.

Investigation of biochemical parameters is useful, yet often limited to smaller patient samples (e.g., dozens of patients in all the papers cited above), whereas questionnaires can readily be used in a wider segment (e.g., hundreds in Pia-Mara Wippert et al., and thousands in Hüfner et al.). In addition to the material and labor costs of biochemical tests, patient willingness is also a limiting factor: only one third of patients with chronic low back pain committed to an additional biochemical test battery even if it could assist in diagnosis (Wippert et al.). Fortunately, questionnaire scales can be highly informative: analyzing the response patterns of stress ratings, Hüfner et al. suggest that these profiles may be linked to published findings on neuroinflammation, levels of vitamin D, gut dysbiosis, and mitochondrial dysfunction. This link points to the importance of incorporating biochemical indicators with scales, to (1) increase diagnostic power, (2) refine scale interpretation by linking symptom profiles to specific underlying conditions, and (3) aid in the faster development of pointed scales for assessment of specific conditions.

In addition to the development of clinically accessible tests, the integration of biochemical parameters with diagnostic procedures is promoted through the understanding that similar symptoms may arise from different molecular mechanisms. This implies that personalized treatment should consider a panel of biochemical markers that could point to a specific underlying molecular origin. To support these approaches, infrastructure in translational medicine should be created, helping the research to address patient needs and new treatments. Matera-Witkiewicz et al. characterize Poland's biobanking network across research infrastructure, quality management, and private-public comparisons, to ascertain quality processes, materials, and data for research and preclinical work that could serve patients world-wide. The piece by Knoppers et al. investigates the rarely-addressed patient and caregiver voices in personalized medicine, exploring the relationship between research and specifically affected communities, such as patients with cystic fibrosis and their caregivers. Findings show that a lifelong progressive and terminal disease stimulates the relationship between the patients/caregivers and researchers, with patients/caregivers considering research intrinsic to their experience and seeking greater access to research results and advancements. Promoting such interactions would foster awareness that the joint effort greatly enriches translation of new therapies and personalized medicine. The communication between patients, caregivers, researchers, and clinicians in designing and implementing research would not only address patient needs, but also feed into advancement through diversity in research.

While there have been significant advances in the technologies and standards involved in storing and analyzing human samples, certain types of questions cannot be answered using human cells or organoids, but require animal experiments due to complex regulation of physiological functions by subordination and interaction of different cells/tissues in the organism. Translational medicine *via* animal models is presented in four of the 13 papers in this collection, which are dedicated to molecular mechanisms of pathologies and/or their therapies. Boyko et al. study a recently discovered *DHTKD1*-encoded enzyme whose expression is associated with obesity and diabetes, but the underlying mechanisms are unknown. They show that the enzyme impacts biological function through protein glutarylation and that pyruvate dehydrogenase complex is among the glutarylated proteins. As the complex is essential for glucose oxidation, its *DHTKD1*-dependent glutarylation may explain *DHTKD1* involvement in diabetes. Yang et al. address molecular mechanisms of the osteogenesis stimulation by the widely used hypoglycemic drug metformin. This drug has potential therapeutic effects in both the diabetes-induced and postmenopausal osteoporosis. The researchers reveal molecular markers and pathways involved in the metformin-induced osteogenic differentiation, which may be useful for personalized medicine to control the different actions of metformin. Two papers of the collection deal with exaggerated systemic inflammation, which has drawn extensive attention during the recent pandemic. Müllebner et al. aim at dissecting specific pathways during the exaggerated systemic inflammation, leading to tissue damage in a model of peritonitis and its surgical treatment. The authors reveal a causal relationship between unfolded protein response and onset of systemic inflammation, which must be accounted for in therapeutic approaches to prevent liver damage. A “cytokine storm” is a key feature of the exaggerated inflammation observed in SARS-CoV-2 infection and sepsis. *In vitro* and *in vivo* models are used by Schapovalova et al. to demonstrate beneficial effects of a herbal treatment on the excessive pro-inflammatory responses associated with changed blood formula and “sickness behavior”. Such herbal compositions are intensely studied within the PhytoAPP project supported by the European Union's Horizon 2020 research and innovation program, as they offer affordable solutions in communities that are in need of stronger healthcare systems.

Through this collection findings, insights, and future directions, we would like to highlight the importance of diversity in research across any dimension, including ethnicity, age, and health status. We offer our thanks to the participants, authors, reviewers, and editors involved in this collection to promote women in translational medicine. We look to the diversity of current and future work in contributing to the highest quality of research in health science and translation.

## Author contributions

All authors listed have made a substantial, direct, and intellectual contribution to the work and approved it for publication.

## Funding

VB acknowledges financial support by RFBR grant 20-54-7804.

## Conflict of interest

The authors declare that the research was conducted in the absence of any commercial or financial relationships that could be construed as a potential conflict of interest.

## Publisher's note

All claims expressed in this article are solely those of the authors and do not necessarily represent those of their affiliated organizations, or those of the publisher, the editors and the reviewers. Any product that may be evaluated in this article, or claim that may be made by its manufacturer, is not guaranteed or endorsed by the publisher.
